# The Annual Cost of a Doctor's Motor-Car

**Published:** 1911-05-27

**Authors:** 


### Motoring Notes.

### THE ANNUAL COST OF A DOCTOR'S MOTOR-CAR.

In a former article I laid stress upon the advisability of obtaining a car of the smallest power which would do work efficiently, and to have greater regard to the working expenses than to the first cost of the car, provided one of the best makes is obtained.

In estimating the cost of motoring when considering the purchase of a car, the prospective buyer is led away by advice given by well-meaning friends, who suggests cars of fifteen horse-power and more and with multiple cylinders. He is alarmed by warnings of failure to climb hills, and the noise that such a car makes when travelling. It is very tempting to go at a speed of thirty or forty miles an hour, but to a medical man this is quite unnecessary, and it is also unpleasant to attend the court to be fined for exceeding the speed limit; beside all this, such speed has to be heavily paid for in petrol and tyres. My own experience of one year's running with its cost might be interesting. It will be noticed that a very small allowance is made for the cost of cleaning. This is due to the cleaning being carried out by an inexperienced lad, and I have no doubt that, although he has made the car look clean enough, his inexperience has led to some deterioration of the varnish. This varnish, although not very bad, shows some minute cracks, and as a matter of precaution I am having the car re-varnished and retouched.

What one hears of the enormous tyre bills that motoring friends have to pay, is apt to frighten the prospective buyer, but with discretion in selection (and here again the best is the cheapest) and careful driving and attention, this item can be reduced to a minimum. My own car has now run one year. It is a De Dion Bouton eight horse-power single-cylinder car, fitted with a double-seated body with hood, and the weight is about 15 cwt. Tyres are Dunlop grooved 760 mm. by 90 mm., with extra-heavy treads. In addition to professional work in a somewhat hilly country, I have taken several short tours. On a straight long run my consumption of petrol has been forty-two miles to the gallon, but naturally this low consumption cannot be maintained over a year's professional work with constant stopping and starting and frequently running on second-speed.

The running condition of the car is at the present time better than when new. The following figures are taken from carefully-kept accounts. The petrol has been obtained from one firm and all vouchers have been kept. My original estimate was £50 for one year's running. No allowance has been made for depreciation or interest on capital. Miles run, 4,884:—

| | £ | s. | d. |
|---|---|---|---|
| Tyres, new (two covers on back wheels) | 9 | 0 | 0 |
| Tyres, retreading (two retreaded covers to front wheels) | 4 | 14 | 2 |
| Petrol, 146 gals. at 1s. | 7 | 18 | 2 |
| Lubricating oil | 1 | 6 | 0 |
| Waste and cleaning materials | 0 | 3 | 6 |
| Washing car (boy at 1s. 6d. per week) | 4 | 0 | 0 |
| Repairs (nil) | | — | |
| Tax and driving licence | 1 | 6 | 0 |
| Garage and cleaning away from home | 0 | 17 | 6 |
| Re-varnishing and re-touching paint | 3 | 8 | 6 |
| Sparking plugs | 0 | 10 | 0 |
| Lighting | 0 | 5 | 0 |
| Rubber paint | 0 | 2 | 0 |
| Changing tyre covers at garage | 0 | 8 | 0 |
| Insurance | 7 | 17 | 6 |
| | £41 | 16 | 4 |

This works out at 2.05 pence per mile for all expenses, which is remarkably economical. The mileage per gallon of petrol works out on the whole running at 33.45 miles per gallon.

I may add that I very frequently have four people in the car, and can travel at twenty-five miles per hour on the level, and have not yet met the hill that the car cannot climb.

It must be understood that the two new covers for the back wheels are not to replace ones that are completely worn out, but as these covers were getting a little thin on the treads, the original new cover was taken from the Stepney wheel and put on the back wheel, and it is only fair to charge this to running account, and a new cover purchased and put on the other back wheel. Both the old covers are fit for re-treading, with a further life of about 3,000 miles. One of them has been put on the Stepney wheel and the other makes a spare tyre.

